# Beyond blood gases: diaphragm-guided sequential non-invasive ventilation for COPD with type II respiratory failure

**DOI:** 10.3389/fmed.2026.1928016

**Published:** 2026-08-24

**Authors:** Yige Wang, Lu Yao, Qiang Xiao, Yafen Tan

**Affiliations:** 1 Second Clinical Medical College, Nanjing Medical University, Nanjing, Jiangsu, China; 2 Department of Pulmonary and Critical Care Medicine, Changde Hospital, Xiangya School of Medicine, Central South University (The First People’s Hospital of Changde City), Hunan, China

**Keywords:** AVAPS, COPD, deventilation syndrome, diaphragm, diaphragmatic thickening fraction, NAVA, noninvasive ventilation, type II respiratory failure

## Abstract

Chronic obstructive pulmonary disease (COPD) with type II respiratory failure is driven by diaphragm load–capacity imbalance and abnormal central respiratory drive, yet clinical decision-making for sequential non-invasive ventilation (NIV) remains largely dependent on blood gas normalization. This narrative review, informed by a structured literature search of PubMed, Embase, and Cochrane Library (January 2000 to June 2026), synthesizes physiological evidence supporting a shift from blood gas–guided to diaphragm–guided sequential NIV management. We propose a testable, three-tier conceptual framework stratified by bedside-measured diaphragmatic thickening fraction (DTF): patients with DTF below 20% may experience severe diaphragmatic impairment requiring sustained high-intensity spontaneous/timed ventilation; those with DTF between 20 and 30% likely retain limited reserve suited to adaptive average volume-assured pressure support as a transitional bridge; and patients with DTF above 30% presumably possess sufficient muscle recovery to transition to proportional assist ventilation or neurally adjusted ventilatory assist for diaphragmatic conditioning. These DTF cutoffs and corresponding mode allocations are provisional hypotheses extrapolated from acute NIV and ICU weaning cohorts and require prospective multicenter validation before routine clinical use. This work addresses three key evidence gaps: longitudinal peri-transition DTF trajectories, ventilation mode comparisons with diaphragmatic functional endpoints, and unconfirmed stratification thresholds, with dedicated prospective trial designs proposed accordingly. Importantly, conventional blood gas assessment remains the validated core standard of care, and this DTF-based strategy only serves as an adjunct physiological tool. Combined serial diaphragmatic ultrasound and respiratory drive monitoring shifts clinical focus from passive biochemical correction to comprehensive physiological recovery, delivering an individualized, physiology-centered strategy to mitigate ventilatory transition failure in advanced COPD.

## 1 Introduction

About 300 million people around the world suffer from chronic obstructive pulmonary disease (COPD) (1), which is the third leading cause of death globally (2). In 25%–30% of patients with advanced disease, chronic hypercapnic respiratory failure (type II respiratory failure) occurs (3), bringing an annual mortality rate of 20%–30% (4). Based on current knowledge, the pathophysiology of type II respiratory failure is determined not simply by impaired gas exchange, but rather by diaphragm load-capacity imbalance and central respiratory drive abnormalities (5, 6). Dynamic hyperinflation contributes to diaphragm flattening and sarcomere shortening, decreasing contractile efficiency. Hypercapnia further lowers the already attenuated respiratory drive, forming a vicious cycle that maintains ventilatory failure (7, 8).

Noninvasive ventilation (NIV) is the primary method of treatment for acute exacerbations and stable COPD disease (9–11). High-intensity pressure support unloads fatigued diaphragms and corrects hypercapnia during acute respiratory acidosis. In the stable phase, lower-intensity support sustains alveolar ventilation and reduces disease exacerbation frequency. The optimal approach for sequential hospital-to-home NIV transition remains largely empiric and has not been fully standardized (12, 13). This transition is particularly difficult, as patients’ respiratory status shifts continuously after acute episode resolution, with diaphragmatic function recovering slowly over time (14).

Current clinical decision-making mainly relies on blood gas parameters. Emerging evidence reveals two important caveats of this conventional strategy. First, restoration of normal blood gas levels does not equate to the recovery of diaphragmatic function (15). Second, this method fails to account for physiological variation among individual patients. One study reported that approximately 58% of COPD patients develop deventilation syndrome following NIV withdrawal, which manifests as acute dyspnea, oxygen desaturation and dynamic hyperinflation (16). This indicates that poor diaphragmatic functional reserve, rather than persistent hypercapnia, serves as the key contributor to sequential therapy failure (5).

Sequential therapy is essentially a transition from diaphragmatic “rest” to diaphragmatic “exercise.” Different NIV modes including S/T (spontaneous/timed), AVAPS (average volume-assured pressure support), and PAV/NAVA (proportional assist ventilation/neurally adjusted ventilatory assist) differ in their capacity to modulate this physiological transition in principle (17, 18). Existing studies, based on current evidence, predominantly adopt gas exchange or clinical events as research endpoints, with limited attention paid to diaphragmatic function and respiratory drive as physiological targets. Therefore, evidence-based guidance for NIV mode selection in sequential therapy remains insufficient.

The objectives of this review are to: (i) summarize the physiologic mechanisms of diaphragmatic function and respiratory drive in COPD-related type II respiratory failure; (ii) compare the differential effects of S/T, AVAPS and PAV/NAVA modes on diaphragmatic unloading and respiratory drive; (iii) define the critical evidence gaps regarding hospital-to-home NIV transition; and (iv) propose a diaphragm-guided approach for sequential mode selection, and offer future research directions to validate this clinical strategy.

Literature search strategy: This narrative review was informed by a structured literature search of PubMed, Embase, and Cochrane Library databases from January 2000 to June 2026. Search terms included combinations of: “COPD,” “chronic obstructive pulmonary disease,” “noninvasive ventilation,” “NIV,” “diaphragm,” “diaphragmatic thickening fraction,” “DTF,” “diaphragmatic ultrasound,” “respiratory drive,” “P0.1,” “NAVA,” “AVAPS,” “S/T,” “sequential therapy,” “weaning,” and “deventilation syndrome.” No language restrictions were applied. Additional references were identified by manual screening of bibliographies of retrieved articles and relevant reviews. Literature screening was performed by two authors independently, with disagreements resolved through discussion with a third author. Priority was given to randomized controlled trials, systematic reviews, meta-analyses, and prospective cohort studies with clear methodology.

## 2 Diaphragm function and respiratory drive in COPD-related type II respiratory failure: pathophysiology and therapeutic implications

### 2.1 Diaphragmatic dysfunction: from pathology to clinical assessment

The diaphragm is the chief inspiratory muscle, contributing around 70% of the generation of tidal volume during quiet breathing (19). In advanced COPD, chronic dynamic hyperinflation places the diaphragm at a severe mechanical disadvantage: inspiratory lung expansion shortens and flattens diaphragmatic muscle fibers, shifting the muscle to the inefficient segment of its length-tension curve (20, 21). Long-term adaptive sarcomere loss further diminishes contractile force (22), jointly elevating the risk of ventilatory decompensation during acute exacerbation or exertion (23, 24).

During acute exacerbation of COPD, elevated airway resistance and expiratory flow limitation trigger severe dynamic hyperinflation and intrinsic positive end-expiratory pressure (PEEPi), creating an inspiratory threshold load that drastically increases breathing work. Static respiratory mechanics and transdiaphragmatic pressure were assessed in a physiological study of 12 AECOPD patients who failed NIV. Patients not responding to NIV had significant diaphragmatic dysfunction, with the majority exhibiting transdiaphragmatic pressure values below 20 cmH₂O (25). Static lung elastance correlated positively with transdiaphragmatic pressure, whereas dynamic PEEPi correlated inversely, suggesting that mechanical derangements directly decrease diaphragmatic contractility (26).

Diaphragmatic ultrasound is now becoming a useful and objective bedside tool to evaluate diaphragm function (27). Three core metrics are applied clinically: diaphragmatic excursion (reflecting tidal volume capacity), diaphragmatic thickening fraction (calculated as [inspiratory thickness − expiratory thickness]/expiratory thickness × 100%, correlated with transdiaphragmatic pressure), and thickening ratio for supplementary reserve evaluation (28). Spontaneous breathing diaphragmatic thickening fraction below 20% is widely accepted as a marker of clinically meaningful diaphragmatic impairment in critically ill and acute exacerbation populations (29). However, it is important to note that this threshold was derived from acute NIV-failure and ICU-weaning studies; its applicability to the post-recovery transition setting remains unconfirmed and requires prospective validation.

Multiple cohort studies have validated the clinical utility of DTF. A prospective study of 41 AECOPD patients found that 24.3% of participants had a DTF below 20%, which was strongly associated with NIV failure, prolonged intensive care unit stay, and increased tracheostomy requirements (26). An emergency department study of 60 patients further demonstrated that DTF below 20% predicted NIV failure with 84.6% sensitivity and 91.5% specificity (30). A larger prospective study of 111 patients reported that DTF measured 2 hours after NIV initiation predicted 48-h treatment failure with an area under the receiver operating characteristic curve of 0.94 (31). Additionally, a study of 94 AECOPD patients confirmed DTF as an independent predictor of NIV clinical outcomes (32). Recently, a multicenter emergency department cohort demonstrated that diaphragmatic dysfunction assessed by point-of-care ultrasound is an independent predictor of prolonged mechanical ventilation, further supporting the translational value of bedside diaphragm assessment in acute respiratory care (33).

**Figure 1:**
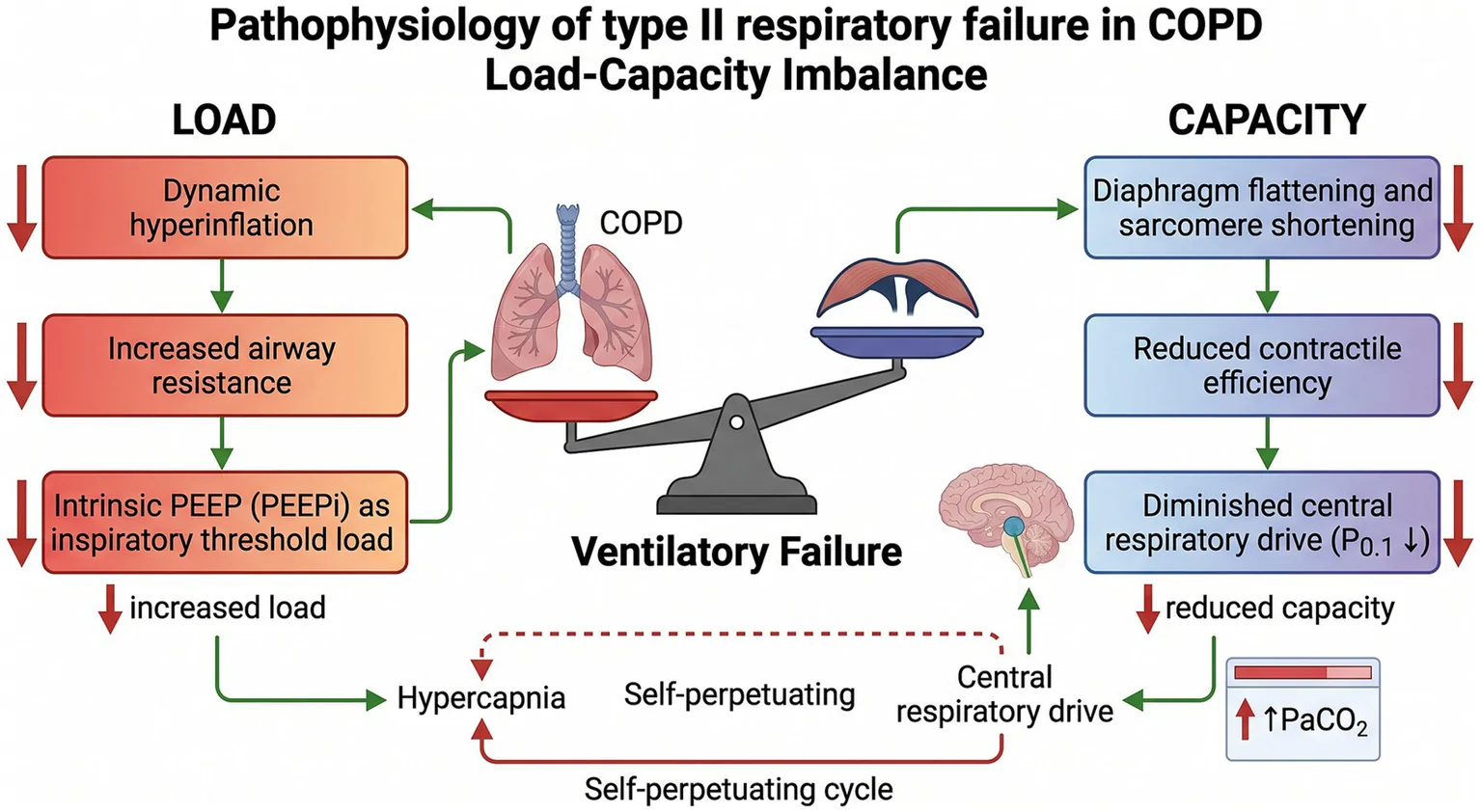
Pathophysiology of type II respiratory failure in advanced COPD: load-capacity imbalance and the vicious cycle of ventilatory failure. (In patients with advanced COPD, chronic dynamic hyperinflation, increased airway resistance, and intrinsic positive end-expiratory pressure (PEEPi) collectively elevate respiratory load (left panel). Concurrently, diaphragm flattening, sarcomere shortening, and diminished central respiratory drive (reflected by reduced P0.1) reduce neuromuscular capacity (right panel). When load persistently exceeds capacity, ventilatory failure ensues. Hypercapnia further suppresses central respiratory drive (red dashed inhibitory arrow), creating a self-perpetuating cycle that sustains ventilatory failure. PEEPi, intrinsic positive end-expiratory pressure; P0.1, airway occlusion pressure at 0.1 s; PaCO_2_, partial pressure of carbon dioxide).

Despite its widespread clinical utility, DTF interpretation carries inherent limitations. DTF is highly effort-dependent, and elevated elastic or resistive inspiratory loads can increase DTF while reducing diaphragmatic excursion, creating discordant readouts (34). Measurement reliability is operator-dependent, with moderate inter-rater (ICC 0.774–0.781) and intra-rater (ICC 0.789–0.817) reproducibility (35). Practical barriers further complicate widespread adoption, including training requirements, poor acoustic windows in patients with obesity or hyperinflation, and substantial heterogeneity in optimal DTF cutoffs across studies (30%–36%) (27). For instance, one study from the same group reported an optimal DTF cutoff of approximately 42% for identifying successful weaners in a larger cohort (36), whereas an earlier study did not report a specific cutoff (32), further illustrating the variability in threshold estimates. These limitations indicate that DTF should not be interpreted in isolation but within full clinical context, and that all proposed thresholds remain provisional until validated in prospective studies (see Figure 1).

### 2.2 Central respiratory drive: 0.1 s airway occlusion pressure as a neglected therapeutic target

Central respiratory drive refers to the output from medullary respiratory centers to respiratory motor neurons. It integrates afferent signals from peripheral and central chemoreceptors, mechanoreceptors, and higher cortical centers (37). Evaluation of respiratory drive provides important insight into patients’ intrinsic ventilatory capacity and chemosensitivity, yet this assessment remains underutilized in NIV-related clinical decision-making (38).

Airway occlusion pressure at 0.1 s (P0.1) is the most commonly adopted bedside assessment index. It is measured via brief airway occlusion at the onset of inspiration, recording the pressure generated within the initial 100 milliseconds of inspiratory effort. As this measurement is acquired prior to inspiratory flow initiation, it is relatively independent of respiratory mechanics and conscious voluntary control (39). In healthy spontaneously breathing adults, P0.1 is typically below 1.5 cmH₂O (40). In the clinical setting of mechanical ventilation, values below approximately 1.5 cmH₂O suggest diminished central respiratory drive, which may occur in the context of sedation, metabolic alkalosis, or central hypoventilation syndromes (39). Values exceeding 3.5–4 cmH₂O reflect elevated respiratory drive, typically stemming from insufficient ventilatory support or increased respiratory load (39), whereas values above 6 cmH₂O denote markedly excessive drive that is associated with dyspnea and increased mortality (41). Emerging clinical evidence indicates that this validated assessment tool has not been widely implemented in routine clinical practice (42).

A prospective study of 88 subjects diagnosed with COPD or obesity-hypoventilation syndrome receiving positive airway pressure therapy identified significantly elevated P0.1 levels in patients with air trapping. After 6 months of standardized treatment, P0.1 levels decreased in the air trapping group, confirming that effective intervention can normalize excessive respiratory drive (43). This normalization of P0.1 after treatment aligns with previous reports of reduced respiratory drive under effective ventilatory support.

Respiratory drive physiologically declines during sleep, particularly during rapid eye movement (REM) sleep, due to reduced chemosensitivity (44). In COPD patients with pre-existing impaired respiratory mechanics, this reduction leads to severe nocturnal hypoventilation (45). REM-related hypoventilation serves as a major contributor to diurnal hypercapnia in COPD populations. Overnight carbon dioxide retention elevates serum bicarbonate levels, which further blunts wakefulness chemosensitivity and forms a self-perpetuating cycle of hypercapnia and reduced ventilatory responsiveness (46).

Pharmacological agents can further suppress central respiratory drive. A retrospective analysis of 339 COPD patients receiving long-term NIV demonstrated that benzodiazepine use was associated with reduced survival rates, while opioid use correlated with increased baseline breathlessness (47). Respiratory depressant medications carry potential clinical risks, particularly during the peritransition period, when patients’ respiratory functional reserve is already compromised.

### 2.3 From blood gas normalization to diaphragm-drive optimization: a paradigm shift

Normalization of blood gases does not indicate diaphragmatic recovery. In a study of 30 patients with severe COPD and chronic hypercapnic respiratory failure followed for 12 months on high-intensity home NIV, PaCO₂ improved significantly. However, the absolute increase in DTF was only 14%, and the majority of patients still had DTF values below normal levels (48). In 25 hypercapnic COPD patients, transdiaphragmatic pressure remained unchanged after 2-month NIV despite good compliance and significant PaCO₂ reduction (49). The recovery of blood gases and diaphragmatic function are not synchronous. This asynchrony between biochemical normalization and diaphragmatic recovery may stem from the patient’s intrinsic central drive resetting at higher baseline PaCO₂ levels, a physiological adaptation that conventional blood gas monitoring fails to capture. Relying solely on blood gas indicators may lead clinicians to prematurely reduce ventilatory support for patients whose diaphragms have not yet recovered sufficient functional reserve (48). Nevertheless, blood gas monitoring combined with clinical assessment remains a valid standard of care validated by numerous clinical trials. The diaphragm-guided framework proposed herein serves as a supplementary, rather than replacement, decision tool to identify transition-failure high-risk patients.

NIV serves different purposes at different treatment stages. During acute exacerbation, the core goal is respiratory unloading: minimizing breathing workload, resting the fatigued diaphragm, and rapidly correcting systemic acidosis (29). When transitioning to home NIV, the treatment focus shifts to functional training, which maintains diaphragmatic activity while avoiding muscle overload and gradually builds diaphragmatic reserve (50). Current clinical strategies fail to clearly differentiate these two functional orientations in theoretical and practical operations.

Multiple clinical studies have consistently verified that a DTF below 20% predicts NIV failure (51). Based on this evidence, we propose the “diaphragmatic functional threshold” hypothesis. Conceptually, a DTF value lower than 20% represents severe impairment of diaphragmatic contractile reserve (31). Patients with persistent DTF below this threshold may require sustained high-intensity NIV treatment, mostly with S/T ventilation mode. When DTF ranges from 20% to 30%, the diaphragm acquires limited but available reserve to start the weaning transition process. Emerging evidence suggests that adaptive unloading modes such as AVAPS can achieve a smoother treatment transition at this stage (52). For patients with DTF exceeding 30%, diaphragmatic functional reserve may indicate relatively preserved function, and diaphragmatic activity-preserving modes including PAV and NAVA become feasible treatment options (53). All stratification boundaries are provisional and require multicenter prospective validation before routine clinical implementation.

The application value of this framework extends far beyond simple ventilation mode selection. Firstly, sequential therapy should be an individualized and tailored intervention, guided by serial diaphragmatic ultrasound assessments rather than a one-size-fits-all protocol (54). Secondly, this framework provides a reasonable physiological explanation for deventilation syndrome. Patients who develop DVS after NIV withdrawal generally have DTF levels below the critical threshold required to sustain independent spontaneous breathing (14). Thirdly, it supports the clinical application of step-down treatment strategies, which reduce pressure support gradually over days to weeks to allow continuous diaphragmatic conditioning, instead of implementing abrupt ventilatory withdrawal (55).

A number of practical clinical questions remain unresolved. What is the optimal monitoring frequency of diaphragmatic ultrasound during the transition period? Can respiratory therapists and bedside nurses perform accurate and reliable DTF measurements? How do confounding factors such as nutritional status, corticosteroid administration, and comorbid conditions affect the correlation between DTF level and weaning outcomes? These questions must be resolved before the diaphragm-guided ventilation strategy can be widely generalized in clinical practice. This hypothesis still needs prospective clinical validation, yet it provides a testable new framework that shifts the traditional clinical focus from simple gas exchange improvement to comprehensive physiological recovery (56).

## 3 Differential physiological effects of noninvasive ventilation modes in COPD with type II respiratory failure

### 3.1 S/T mode: fixed support, fixed limitations

The spontaneous/timed (S/T) mode is the most widely adopted noninvasive ventilation mode in global clinical practice. It provides stable inspiratory positive airway pressure (IPAP) on the basis of constant expiratory positive airway pressure (EPAP). The pressure support level received by patients is determined by the gradient between IPAP and EPAP (57). Such operational simplicity makes S/T mode the default ventilation choice in most clinical environments (58).

The physiological effects of S/T mode on diaphragmatic function are well-established but have important limitations. Fixed pressure support reduces the transdiaphragmatic pressure required to generate tidal volume, thereby effectively unloading inspiratory muscles (59). A clinical study recruiting 10 stable hypercapnic COPD patients on home mechanical ventilation confirmed that high-pressure ventilation settings (mean IPAP 25 cmH₂O) led to more significant reduction in diaphragmatic electromyographic activity compared with spontaneous breathing status (60). However, this fixed support cannot dynamically adapt to patients’ fluctuating respiratory drive or changing respiratory mechanical conditions (61). During periods of heightened respiratory drive, such as sleep-disordered breathing and early morning hours, fixed support may be insufficient, resulting in patient-ventilator asynchrony. In contrast, excessive fixed support during low respiratory drive may cause disuse and deconditioning of the diaphragm (59).

These limitations are particularly prominent in sequential ventilation therapy. In conventional transition protocols, pressure support is reduced in a pre-determined, step-wise manner (55). Patients who are adequately supported at IPAP 20 cmH₂O may receive insufficient assistance at IPAP 15 cmH₂O if their diaphragmatic reserve fails to recover adequately (54). Furthermore, S/T mode cannot provide real-time monitoring data on diaphragmatic effort and respiratory drive (62). Clinicians can only judge these physiological conditions indirectly through respiratory rate, accessory muscle participation, and blood gas analysis results.

Despite these limitations, S/T mode remains the standard of care for acute COPD exacerbations and stable hypercapnic COPD patients who require high-intensity ventilatory support (63). Under the diaphragm-guided treatment framework, S/T mode is commonly used for patients with DTF below 20% who require sustained ventilatory support (64). Meanwhile, emerging clinical evidence indicates that adaptive and proportional ventilation modes have unique advantages for patients transitioning to low-level ventilatory support (53).

### 3.2 AVAPS mode: adaptive unloading for transitional support

Average volume-assured pressure support (AVAPS) is a hybrid mode that combines pressure-controlled ventilation with a volume-targeting algorithm (65). The clinician presets the target of the tidal volume, and the ventilator automatically adjusts the IPAP value within the range of the preset value to achieve the target. EPAP and backup rate are set manually. This design enables AVAPS to adapt to changes in respiratory mechanics, patient effort and air leaks (66).

The core physiological benefit of AVAPS is its dynamic adaptability to complex clinical conditions. The ventilator can raise the pressure support when the patient’s respiratory drive rises to deliver a target tidal volume, avoiding excessive respiratory effort and diaphragmatic fatigue (67). Conversely, the ventilator can decrease pressure support when the patient’s drive diminishes or when the diaphragm starts to recover spontaneous activity, preventing over-assisted ventilatory support. This “adaptive unloading” is particularly beneficial during the transition from high-level to low-level ventilatory support (67).

Clinical evidence supporting the application of AVAPS in COPD patients has been continuously accumulated. In a randomized trial of 100 patients with AECOPD, AVAPS improved pH and PaCO₂ levels at 6 and 24 h more significantly than S/T mode and reduced hospital stay (67). In 35 patients with insufficient response to fixed-level pressure support NIV (PS-NIV), switching to AVAPS significantly improved PaCO₂. Notably, 92.8% of patients who achieved stable ventilation status on AVAPS could return to fixed-level PS-NIV, indicating that AVAPS acts as a reliable “bridge mode” during the ventilator transition period (68).

However, it is important to note that not all evidence supports AVAPS superiority. A meta-analysis of five studies comparing AVAPS with conventional pressure support modes in stable COPD patients found that AVAPS was not superior to conventional modes in improving blood gas parameters. However, patients receiving AVAPS ventilation obtained significantly better subjective comfort and perceived sleep quality (69). This finding carries important clinical significance, as patient tolerance is a key factor contributing to long-term adherence to home NIV treatment.

Within the diaphragmatic-guided ventilation framework, AVAPS is most suitable for patients with DTF ranging from 20% to 30%. These patients retain sufficient diaphragmatic reserve to initiate the weaning transition, but are not yet capable of complete spontaneous breathing (70). The automatic pressure support adjustment of AVAPS enables gradual, individualized reduction in mechanical unloading alongside diaphragmatic functional recovery, which may lower the clinical risk of deventilation syndrome (59). This proposed application remains hypothesis-driven and requires prospective validation.

### 3.3 PAV/NAVA mode: preserving spontaneous effort

Proportional assist ventilation (PAV) and neurally adjusted ventilatory assist (NAVA) adopt a fundamentally different approach to ventilatory support. Unlike ventilation modes that deliver fixed or volume-targeted pressure, PAV and NAVA provide supportive pressure in accordance with the patient’s real-time continuous respiratory effort (18).

PAV delivers two types of ventilatory assistance: flow assist to reduce respiratory resistive work and volume assist to alleviate elastic work burden. Clinicians preset the percentage of respiratory resistance and elastance to be unloaded, and the ventilator dynamically calculates and outputs the required pressure in real time. PAV functions as an on-demand ventilation system, where the greater the respiratory effort the patient exerts, the more supportive pressure the ventilator provides (18, 71).

NAVA uses a nasogastric tube with electrodes to measure diaphragmatic electrical activity (Edi), a direct measure of neural respiratory drive. The ventilator delivers pressure support strictly proportional to the Edi signal. Since the Edi signal appears earlier than inspiratory flow, NAVA achieves near-instantaneous triggering and cycling. This mechanism effectively eliminates the trigger delays and cycling errors commonly observed in conventional pressure support ventilation modes (18, 72).

Theoretically, both PAV and NAVA share the core advantage of maintaining and training patients’ inherent respiratory drive and diaphragmatic activity (73). Because ventilatory support is strictly proportional to patient effort, patients must actively participate in every breath. This continuous engagement helps prevent diaphragmatic deconditioning, a common complication of complete or near-complete respiratory muscle unloading in fixed and adaptive ventilation modes. For patients transitioning from high-intensity to low-intensity ventilatory support, PAV and NAVA provide a viable physiological pathway to sustain diaphragmatic conditioning without causing muscle overload (74).

Clinical data on PAV and NAVA in COPD patients is growing but remains relatively limited compared with S/T and AVAPS. In 15 severe COPD patients, noninvasive PAV effectively increased tidal volume and reduced inspiratory work. Nevertheless, PAV was associated with significantly prolonged expiratory cycle delay, and a typical “runaway” phenomenon occurred under high assist levels. This indicates that PAV assist parameters require careful clinical titration to prevent excessive ventilatory support (7).

For NAVA, the evidence is more extensive but yields mixed results. A randomized controlled trial enrolling 40 AECOPD patients verified that NAVA significantly reduced patient-ventilator asynchrony events compared with conventional PSV. However, no significant differences were observed in blood gas exchange improvement, NIV failure rate, and hospital length of stay between the two groups (75). Another trial of 76 AECOPD patients found no significant difference in NIV failure rates between NAVA and adaptive support ventilation (ASV), while ASV required far fewer physician manual adjustments within the first 24 h of ventilation (76). A feasibility study of 55 AECOPD patients demonstrated that individualized adaptive support ventilation (ASVi) shortened hospital stay and improved patient comfort, highlighting the practical operational advantages of automated adaptive ventilation modes for acute COPD exacerbation treatment (77). Collectively, these findings suggest that proportional and adaptive modes offer measurable benefits in patient-ventilator synchrony and operational efficiency, but their superiority over conventional modes in hard clinical endpoints such as failure rate or mortality has not been consistently demonstrated.

**Figure 2:**
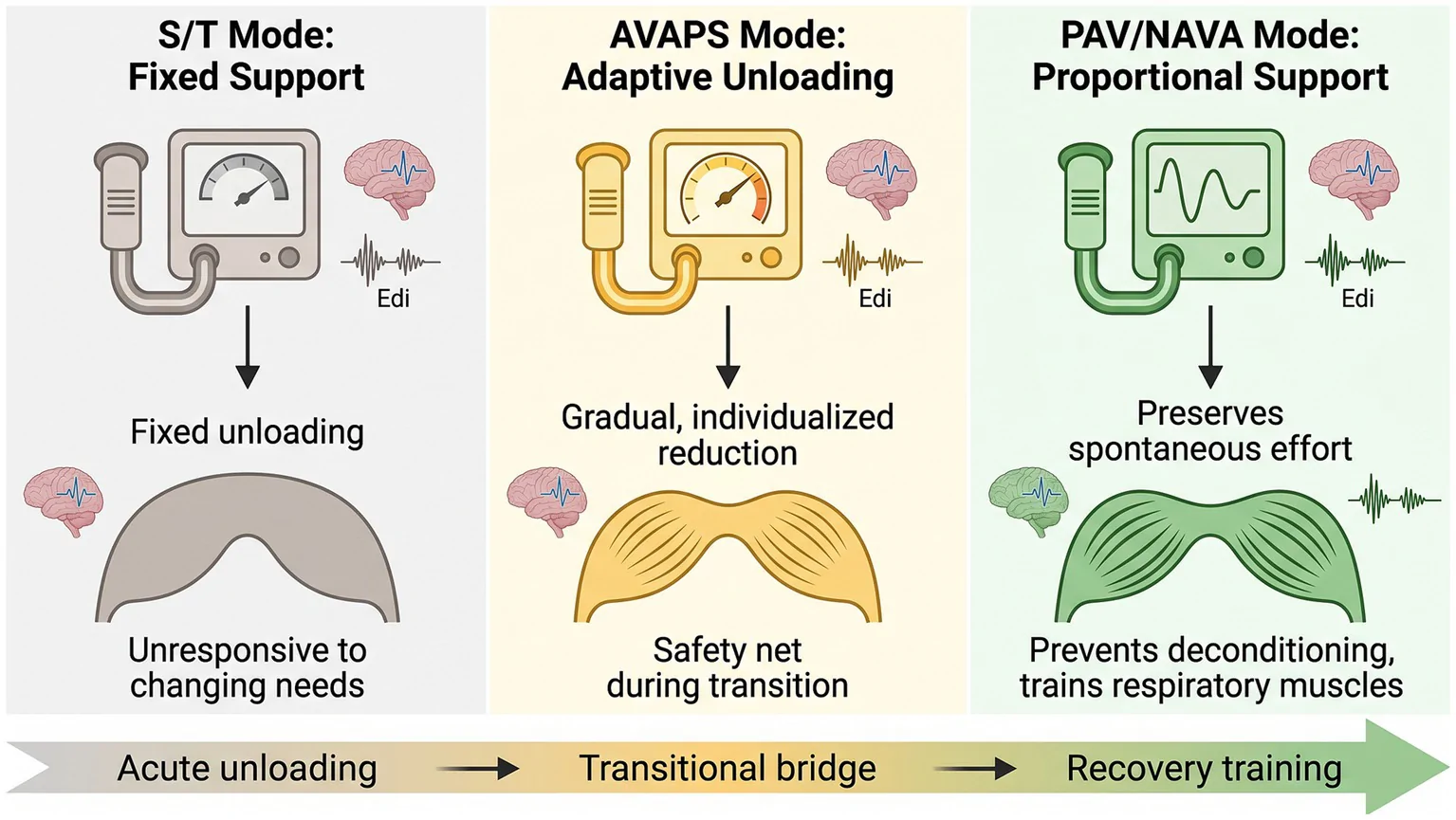
Differential physiological effects of S/T, AVAPS, and PAV/NAVA modes on diaphragmatic function and respiratory drive. (Three noninvasive ventilation modes are compared based on their interaction with the patient’s respiratory drive and diaphragmatic activity. S/T mode (left) delivers fixed pressure support independent of patient effort, providing effective unloading of the inspiratory muscles but no dynamic adaptation to changing respiratory needs. AVAPS mode (center) adapts pressure support to maintain a target tidal volume, offering a gradual, individualized reduction in unloading that serves as a safety net during the transition from high-intensity to low-intensity support. PAV/NAVA modes (right) deliver support proportional to the patient’s real-time respiratory effort, preserving spontaneous diaphragmatic activity, preventing deconditioning, and promoting respiratory muscle training. The gradient timeline at the bottom illustrates the progression from acute unloading through a transitional bridge to recovery training. S/T, spontaneous/timed; AVAPS, average volume-assured pressure support; PAV, proportional assist ventilation; NAVA, neurally adjusted ventilatory assist).

Based on the established principles of diaphragm-protective ventilation, PAV and NAVA are theoretically optimal for patients with DTF above 30%. Because these patients possess adequate diaphragmatic functional reserve, they are ideal candidates for modes that actively preserve and promote respiratory muscle activity (18). Such proportional modes are particularly valuable in the late stage of sequential ventilation therapy, when the clinical goal shifts from muscle unloading to respiratory muscle conditioning (74). However, this recommendation remains hypothesis-driven, as direct evidence linking proportional mode selection to improved diaphragmatic outcomes is still lacking. Furthermore, most commercial home ventilators lack PAV and NAVA functionality, which severely restricts their widespread application in long-term home ventilation (Figure 2).

### 3.4 Comparative summary of the three ventilation modes

The three mode types differ fundamentally in how they interact with the patient’s respiratory drive and diaphragmatic activity. Conceptually, S/T mode delivers fixed support that is independent of patient effort — effective for unloading but unresponsive to changing needs (67). AVAPS mode adjusts pressure support to maintain target tidal volume – it provides a safety net during transition (65). In both PAV and NAVA modes, support is proportional to patient effort, which is physiologically advantageous but technically demanding (18, 73).

The key differences among the three modalities are systematically contrasted in Table 1. Overall, available trial evidence suggests that no single mode demonstrates universal superiority across all clinical endpoints. Consequently, the DTF-linked mode allocation scheme proposed herein lacks direct prospective evidence linking mode choice to diaphragmatic recovery and remains hypothesis-driven. Future randomized stratified trials are required to validate this individualized strategy against universal weaning protocols.

**Table 1:** Comparison of S/T, AVAPS, and PAV/NAVA modes in the diaphragm-guided framework.

Feature	S/T mode	AVAPS	PAV/NAVA
Support basis	Fixed pressure	Volume-targeted adaptive	Effort-proportional
Effect on diaphragm	Fixed unloading	Adaptive unloading	Proportional unloading
Preservation of spontaneous effort	Low	Moderate	High
Dynamic adaptability	None	Good (to tidal volume)	Excellent (to effort)
Patient-ventilator synchrony	Moderate	Good	Best
Evidence base for COPD	Extensive	Moderate	Limited
Availability on home ventilators	Universal	Increasing	Limited
Hypothesized DTF range	<20%	20–30%	>30%
Role in sequential therapy	Acute unloading	Transitional bridge	Recovery training

## 4 Sequential therapy in COPD with type II respiratory failure: physiological challenges and current evidence gaps

### 4.1 Current sequential strategies: a critical review

The transition from high-intensity, hospital-based NIV during acute exacerbation to low-intensity, home-based NIV for chronic management is known as sequential therapy. This transition is likely critical for maintaining stable ventilation and preserving diaphragmatic function after discharge, thereby reducing the risk of subsequent respiratory failure in COPD patients (78, 79).

Evidence on sequential therapy is limited and derives mostly from studies on weaning from invasive mechanical ventilation (IMV). A meta-analysis of 16 randomized trials with 994 patients (most of whom were COPD patients) showed that early extubation plus NIV significantly reduced mortality, ventilator-associated pneumonia, and the duration of invasive ventilation compared to conventional invasive ventilation weaning (80). These data led to the introduction of the “pulmonary infection control window” — a clinical indicator suggesting that pulmonary infection is under control — which was largely adopted clinically as the time of switching (81, 82).

However, these strategies were created for weaning following a period of invasive ventilation, not for switching between noninvasive modes or for hospital-to-home NIV transitions. High-quality research on hospital-to-home NIV transition remains scarce (83). Most studies have focused on whether to initiate home NIV at all, rather than whether or how to transition. Randomized trials have evaluated the feasibility of initiating NIV at home following acute exacerbation, with conflicting results (84, 85). Some studies reported improvements in readmission or survival, while others did not. These discrepancies are likely driven by heterogeneity in patient selection, ventilatory strategy, and transition protocol rather than a lack of treatment effect, although this interpretation remains a mechanistic hypothesis (86).

Current approaches have one major drawback as they essentially use normalization (balance) of blood gases and clinical stability as the key indicators for transition (87). Patients may maintain normocapnia and clinical stability despite persistent diaphragmatic weakness, rendering them vulnerable to ventilatory decompensation following withdrawal of respiratory support. This may account for the variability of patient’s transition to home NIV when some patients achieve success and others end up having recurrent hypercapnia or deventilation syndrome (88). A major evidence gap is the absence of physiological markers that indicate diaphragmatic readiness for transition (89).

### 4.2 Deventilation syndrome: a warning sign of sequential failure

Deventilation syndrome (DVS) is characterised by acute dyspnoea occurring within minutes to hours after NIV discontinuation in patients with chronic hypercapnic COPD. Affected patients present with sudden-onset dyspnea, tachypnea, oxygen desaturation and increased accessory muscle use. If severe, dynamic hyperinflation progresses, and certain patients need NIV reinstitution and/or hospitalization. The syndrome develops usually within the first hour of withdrawal from NIV and can last for 30 min to several hours (16, 88).

A prospective observational study of 67 severe COPD patients reported a prevalence of 58%. However, this single-study estimate should be interpreted with caution, as prevalence varies substantially depending on the definition used and the patient population studied (14, 16). A systematic scoping review identified five studies examining DVS in COPD patients on long-term home NIV, with substantial heterogeneity in definitions and methodology (14). Compared with patients free of DVS, individuals with DVS demonstrate worsened airway obstruction, hyperinflation, elevated PaCO₂ retention, and impaired quality of life and exercise capacity.

A complete understanding of the pathophysiology of DVS is not yet available, but a few mechanisms have been suggested. One hypothesis is that high inspiratory pressures during NIV may acutely improve ventilation but also induce dynamic hyperinflation. A sudden withdrawal of NIV can result in rapid rise of the elastic load which can be greater than the patient’s diaphragmatic capacity (90). Another theory is that NIV-ventilator asynchrony during NIV can result in ineffective triggering and subsequent unloading of the respiratory muscles not maintained after NIV removal (91).

The high risk of DVS may have relevant implications for sequential therapy. It implies that normalizing the blood gas level is not enough to decide when a patient is ready for withdrawal from NIV. Patients who develop DVS likely have DTF below the threshold required to sustain spontaneous breathing without support. This observation directly highlights the need for physiological markers, such as DTF, to guide transition decisions (36).

### 4.3 Diaphragm dynamics during the transition period: an unstudied domain

The hospital-to-home NIV transition is a time of great physiologic transformation. In acute exacerbation, high-intensity NIV provides near-complete diaphragm unloading, allowing diaphragmatic recovery from fatigue and resolution of dynamic hyperinflation. Once the patient stabilizes, ventilatory support is gradually weaned. The activity of the diaphragm must gradually ramp up to keep ventilation sufficient. Sequential therapy fundamentally represents this transition from diaphragmatic “rest” to “exercise” (92).

To date, however, there are no studies exploring diaphragmatic function during the peritransition period, that is, 48 to 72 h before and after hospital-to-home NIV transition (93). Several fundamental questions remain unanswered. What is the change in DTF during this time? Does it rise gradually with progressive diaphragmatic improvement, or does it become stable? What is the lowest DTF required to maintain unassisted spontaneous breathing? Can early changes in DTF during the peritransition period predict subsequent DVS development? Relevant studies have established DTF thresholds for NIV weaning outcomes, yet the dynamic changes occurring within this narrow timeframe have not been clarified (31, 94).

Related clinical fields provide indirect evidence supporting the utility of diaphragmatic monitoring. In the ICU setting, DTF measured during spontaneous breathing trials predicts successful extubation. A DTF below 20%–30% is associated with an increased risk of weaning failure (95, 96). In the NIV setting, DTF recorded within the first 2 hours of treatment serves as a predictor of NIV failure within 48 h (31). While evidence from ICU and acute NIV settings supports the utility of diaphragmatic monitoring, its direct application to the hospital-to-home transition phase has not yet been investigated.

At present, research dedicated specifically to this transition phase remains limited, forming a notable evidence gap. Future prospective studies are urgently needed to elucidate diaphragm functional variability during the transition period and determine the correlation between DTF thresholds and transition success or failure (54). Such investigations would establish a solid physiological basis for standardized, evidence-based transition protocols.

### 4.4 Physiological analysis of transition strategies

Current clinical practice shows substantial variation in the implementation of hospital-to-home NIV transition. Some clinicians adopt an abrupt withdrawal approach, discontinuing NIV entirely once patients achieve normalized blood gases and clinical stability. Others implement a stepwise reduction strategy, progressively lowering pressure support over days to weeks (97, 98). A randomized trial enrolling 90 AECOPD patients compared immediate pressure support withdrawal with gradual stepwise reduction, finding no significant difference in transition success rates. However, the immediate withdrawal group exhibited significantly shorter total NIV duration and hospital length of stay (97).

These data confirm that abrupt withdrawal is feasible for many patients, but the specific subgroups suitable for this approach remain unclear, as existing trials have not incorporated physiological markers of diaphragmatic function (54, 99). Conceptually, patients with preserved diaphragmatic reserve are more likely to tolerate abrupt discontinuation, whereas those with limited reserve may require a more gradual transition. However, this hypothesis requires prospective validation.

From a physiological perspective, an individualized approach, tailored to diaphragmatic reserve rather than a universal protocol, is theoretically preferable. The mode of ventilation also matters: S/T mode with stepwise pressure reduction requires regular clinician adjustment, AVAPS offers automatic pressure titration as a safety net during the gradual transition, and proportional modes (PAV/NAVA) allow the diaphragm to progressively increase workload without overload (53, 74). Future studies should compare different transition strategies stratified by baseline DTF levels to identify the optimal approach for each patient subgroup.

## 5 Diaphragm and respiratory drive monitoring: navigation tools for sequential therapy

### 5.1 Complementary roles of diaphragmatic ultrasound and respiratory drive monitoring

Diaphragm ultrasound evaluates effector muscle function, while P0.1 and diaphragmatic electrical activity quantify upstream central neural drive, forming complementary assessment tools (100). During pressure tapering, a rising P0.1 signals insufficient diaphragmatic reserve, while stable or reduced values confirm patient adaptation (101). Diaphragmatic electrical activity via specialized nasogastric catheters directly measures neural inspiratory activity and guides neurally adjusted ventilatory assist titration, yet catheter requirements limit routine bedside use (102, 103). Surface parasternal electromyography offers a non-invasive surrogate for diaphragmatic electrical activity but lacks standardized validation for sequential therapy (104, 105).

### 5.2 Integrated diaphragmatic thickening fraction-guided sequential workflow

Based on the above evidence, we propose a diaphragm-guided framework for sequential therapy. This framework incorporates DTF as the main decision variable and P0.1 as a secondary, well-validated bedside index that reflects both diaphragmatic function and neural respiratory drive (39, 64). Crucially, this framework is presented as a testable hypothesis requiring prospective validation, not as an established clinical algorithm.

**Figure 3:**
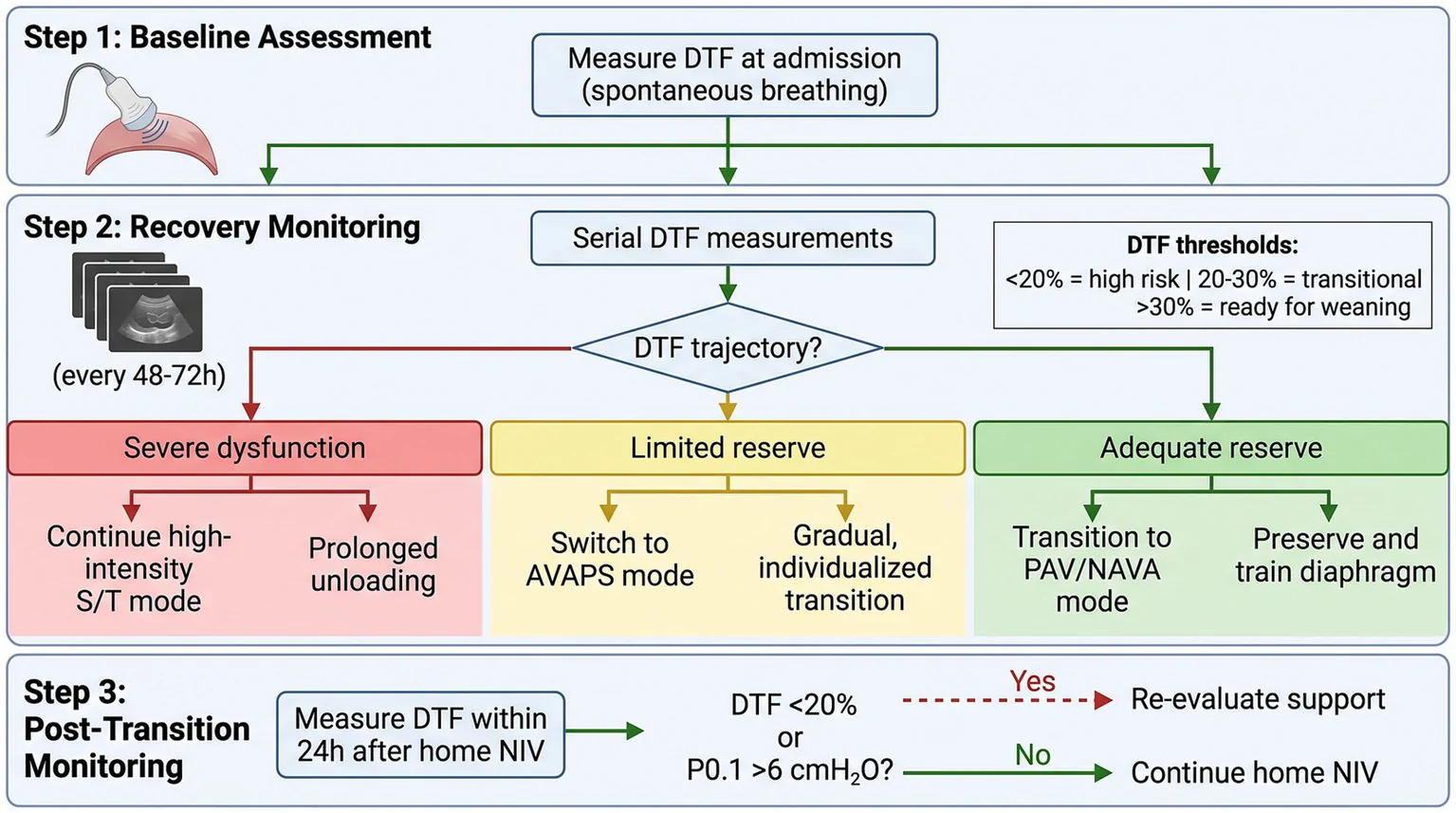
Diaphragm-guided sequential therapy framework based on diaphragmatic thickening fraction (DTF) thresholds. (This proposed framework outlines a three-step assessment pathway and should be interpreted as a testable hypothesis rather than a definitive clinical algorithm. Step 1: Baseline assessment. DTF is measured during spontaneous breathing upon hospital admission, prior to noninvasive ventilation initiation. A DTF below 20% may signal severe diaphragmatic dysfunction and elevated risk of noninvasive ventilation failure; a DTF above 30% may suggest relatively preserved diaphragmatic function. These boundary values remain provisional and require prospective validation. Step 2: Recovery monitoring. Serial DTF measurements are obtained every 48–72 h throughout noninvasive ventilation management. A rising DTF may reflect diaphragmatic recovery and could support consideration of treatment transition; stable or falling DTF may point to persistent dysfunction that merits continued respiratory unloading. Parallel 0.1-s airway occlusion pressure monitoring enables complementary evaluation of central respiratory drive. Step 3: Mode selection and transition. For patients with DTF ranging from 20% to 30% (limited functional reserve), average volume-assured pressure support may enable gradual transition by automatically tapering pressure support alongside clinical improvement. For patients with DTF above 30% (sufficient reserve), direct transition to proportional assist ventilation or neurally adjusted ventilatory assist may be preferable to sustain diaphragmatic activity. For patients with persistently low DTF (below 20%) following 1 week of high-intensity spontaneous/timed ventilation, extended mechanical unloading could be considered. Post-transition monitoring: DTF assessment within 24 h after switching to home ventilator settings. A DTF below 20% or 0.1-s airway occlusion pressure exceeding 6 cmH_2_O may necessitate reassessment of ventilatory support intensity).

As delineated in our proposed algorithmic framework (Figure 3), there are three steps:

Step 1: Baseline assessment. Measure DTF during spontaneous breathing before NIV initiation at hospital admission. DTF below 20% may indicate severe diaphragmatic dysfunction, and patients with this value have the highest risk of NIV failure and may require a longer unloading period before making transition decisions (31, 51). DTF above 30% indicates preserved diaphragmatic function, and these patients may be more amenable to a more rapid transition.
Step 2: Recovery monitoring. Repeat DTF measurements every 48–72 h during NIV treatment. A rising DTF may suggest diaphragmatic recovery and indicates readiness to consider transition. A stable or declining DTF implies ongoing diaphragmatic dysfunction and means that unloading needs to continue. P0.1 should be monitored concurrently to confirm respiratory drive status (39). A falling P0.1 along with stable or improving DTF may confirm adequate ventilatory support and physiological recovery.
Step 3: Mode selection and transition. For DTF 20%–30%, patients have enough reserve to initiate transition; AVAPS may facilitate a smoother transition by automatically reducing pressure support as the patient improves (68). For DTF > 30%, transition to PAV/NAVA may be preferred to maintain diaphragmatic activity and synchrony (53, 74). For persistent DTF < 20% after 1 week of high-intensity S/T, continued unloading is advised, with adjunctive interventions such as nutritional support and inspiratory muscle training (106). Post-transition DTF should be reassessed within 24 h; a DTF < 20% or P0.1 > 6 cmH₂O may necessitate support re-evaluation (14).

This framework is hypothesis-driven rather than fully evidence-based. It integrates established physiological data and single-marker validation results into a unified, testable clinical algorithm (107). However, multicenter validation of this framework is urgently required. Several key unanswered questions remain: What is the optimal DTF monitoring frequency during the transition period? Can DTF threshold values be validated in multicenter clinical studies? Does DTF-guided transition reduce the incidence of deventilation syndrome compared with conventional clinical care? Addressing these questions is a priority for future research to refine and validate this bedside clinical strategy.

## 6 Current limitations and future research directions

### 6.1 Three Core unresolved questions and corresponding trial schemes

The diaphragm-guided framework proposed in this review identifies three major evidence gaps, each requiring dedicated study designs.

Gap 1: Longitudinal dynamics. No studies characterize diaphragmatic thickening fraction changes across the 48–72 h peri-transition window.

Proposed trial: Prospective observational cohort with serial testing on admission, daily acute-phase measurement, and 12-hourly sampling pre- and post-72-h transition window. Primary outcome: peri-transition recovery trajectory; secondary outcomes: deventilation syndrome incidence and prognosis correlation (54, 56).

Gap 2: Head-to-head mode comparisons using diaphragmatic endpoints. Existing trials prioritize blood gas or clinical adverse event endpoints, with almost no analysis measuring functional changes as primary outcomes.

Proposed trial: Randomized controlled superiority trial in diaphragmatic thickening fraction 20%–30% patients comparing average volume-assured pressure support gradual transition versus conventional blood-gas guided spontaneous/timed weaning. Primary endpoint: 72-h deventilation syndrome incidence; secondary endpoints: noninvasive ventilation failure, readmission, patient comfort (14, 66, 108).

Gap 3: Unvalidated stratification thresholds. The 20% cut-off derives from intensive care unit and acute noninvasive ventilation failure cohorts; the 20–30% and above 30% ranges are theoretical extrapolations without prospective transition-cohort validation.

Proposed trial: Stratified mode comparison randomized controlled trial: patients stratified by baseline diaphragmatic thickening fraction (below 20%, 20–30%, above 30%), randomly assigned to spontaneous/timed, average volume-assured pressure support, or proportional assist ventilation/neurally adjusted ventilatory assist. Primary outcome: 72-h diaphragmatic thickening fraction change; secondary: synchrony, subjective comfort and clinical adverse events (109, 110).

Additional diagnostic accuracy trial: Serial pre-transition testing with 72-h deventilation syndrome follow-up, receiver operating characteristic curve analysis to identify optimal universal transition cut-off values.

### 6.2 Technical and clinical translation outlook

Technological advances have made diaphragm-guided sequential therapy feasible. Portable bedside ultrasound devices are becoming more available and training for their use has been incorporated into respiratory care education programs (111, 112). Automated DTF measurement algorithms, in principle, decrease the variability of observers involved in serial monitoring (113). While AVAPS is increasingly available on home ventilators, PAV and NAVA remain largely confined to ICU platforms; the latter require specialized Edi catheters and are not currently offered on commercial home ventilators. This hardware limitation substantially restricts the practical applicability of the PAV/NAVA arm in home settings.

Remote monitoring technologies may help support the post-transition period. Early detection of possible failure to transition is made possible through telemonitoring of ventilator parameters and patient symptoms. Home care could enable functional monitoring after discharge using ultrasound; however, adequate healthcare resources are needed to standardize this approach (114).

There are still a few clinical uncertainties that need to be addressed for optimizing the framework. Other chronic conditions such as heart failure, obesity, and neuromuscular diseases may moderate the relationship between DTF and transition outcomes (115, 116). The criteria for the use of inspiratory muscle training in patients with low-DTF have yet to be established, and emerging evidence indicates improved diaphragmatic function after IM training (117, 118). Future research is also needed to guide the management of patients who fail DTF-guided transition.

**Figure 4:**
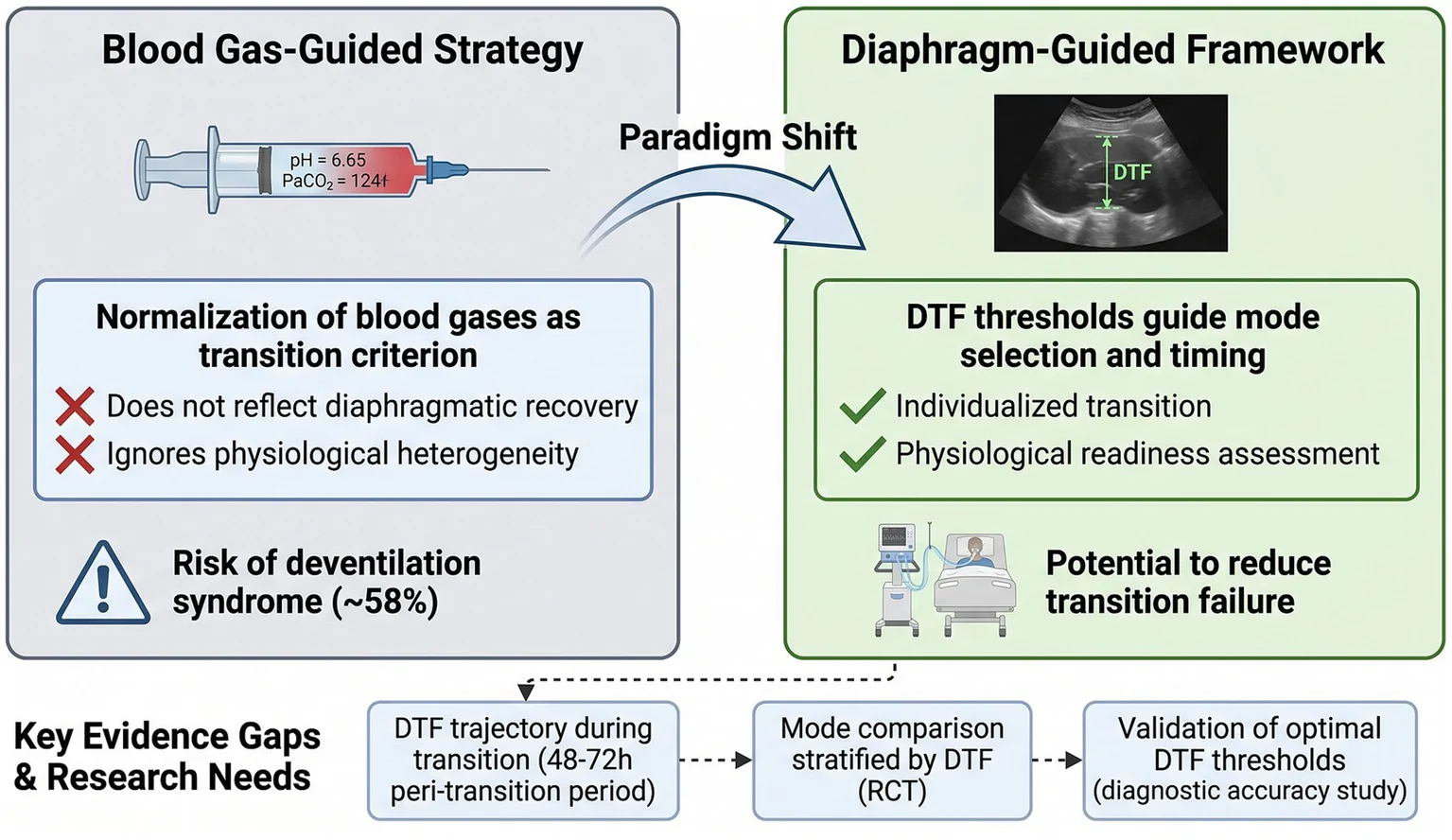
Paradigm shift from blood gas-guided to diaphragm-guided sequential therapy in COPD with type II respiratory failure. Left panel (traditional approach). Clinical decision-making has relied primarily on normalization of blood gases (pH and PaCO₂) as the criterion for NIV withdrawal. However, this strategy has two major limitations: (i) normalization of blood gases does not equate to recovery of diaphragmatic function, and (ii) it fails to account for physiological heterogeneity among patients. Consequently, in one prospective single-centre cohort (*n* = 67), 58% of patients on long-term home NIV met the study’s definition of DVS after termination of nocturnal ventilation; reported prevalence varies substantially with the definition applied. Right panel (proposed diaphragm-guided framework). The framework uses serial diaphragmatic ultrasound measurements of DTF to assess diaphragmatic functional reserve. DTF thresholds (<20%, 20–30, >30%) guide mode selection (S/T, AVAPS, or PAV/NAVA) and transition timing, enabling individualized, physiology-based sequential therapy. Bottom panel (future directions). Three key evidence gaps require prospective validation: (i) the trajectory of DTF during the 48- to 72-h peri-transition period; (ii) mode comparison stratified by DTF in randomized controlled trials; and (iii) validation of optimal DTF thresholds in diagnostic accuracy studies. DVS, deventilation syndrome; DTF, diaphragmatic thickening fraction; NIV, noninvasive ventilation; RCT, randomized controlled trial.

As per available data, this framework should be used as a concept and not as a protocol (31, 54). However, the use of serial diaphragm ultrasound is in addition to blood gas measurements and offers useful physiological data for clinical decision-making (54, 56). The proposed DTF thresholds are preliminary and will need to be validated. Theoretically, the strategy could be used to achieve sequential individualised treatment directed towards the underlying disease pathophysiology in COPD exacerbation patients who develop type II respiratory failure (Figure 4).

## 7 Conclusion

Type II respiratory failure in chronic obstructive pulmonary disease cannot be managed solely through blood gas correction. Its core pathological drivers consist of diaphragmatic dysfunction and impaired central respiratory drive. This review proposes a testable, physiology-centered framework for sequential noninvasive ventilation. Bedside diaphragmatic thickening fraction stratifies the risk of ventilatory decompensation and guides ventilatory mode selection, shifting clinical decision-making from passive biochemical monitoring toward active evaluation of functional recovery.

This study presents a three-tiered individualized weaning model based on diaphragmatic functional status. High-intensity unloading is applied for severe diaphragmatic impairment, adaptive bridging support for patients with limited functional reserve, and effort-proportional conditioning for those with restored diaphragmatic function. This hierarchical strategy provides a physiological foundation for personalized ventilator weaning. Nevertheless, all proposed stratification thresholds remain preliminary and require multicenter prospective validation prior to routine clinical implementation.

Future research should advance beyond current proof-of-concept evidence toward rigorous prospective verification. Key priorities include longitudinal characterization of peri-transition diaphragmatic dynamics, randomized controlled comparisons of ventilation modes based on diaphragmatic functional endpoints, and standardized cut-off identification across heterogeneous patient cohorts. Until robust validation data become available, clinicians should integrate serial diaphragmatic ultrasound with conventional blood gas analysis and general clinical evaluation. Individual weaning trajectories should be interpreted as dynamic physiological recovery processes rather than standardized protocol-driven procedures.

## Glossary

AECOPD: Acute exacerbation of chronic obstructive pulmonary disease
ASV: Adaptive support ventilation
AVAPS: Average volume-assured pressure support
COPD: Chronic obstructive pulmonary disease
DE: Diaphragmatic excursion
DTF: Diaphragmatic thickening fraction
DVS: Deventilation syndrome
Edi: Electrical activity of the diaphragm
EPAP: Expiratory positive airway pressure
ICC: Intraclass correlation coefficient
IMV: Invasive mechanical ventilation
IPAP: Inspiratory positive airway pressure
NAVA: Neurally adjusted ventilatory assist
NIV: Noninvasive ventilation
P0.1: Airway occlusion pressure at 0.1 s
PaCO₂: Partial pressure of carbon dioxide
PAV: Proportional assist ventilation
PEEPi: Intrinsic positive end-expiratory pressure
PSV: Pressure support ventilation
REM: Rapid eye movement
ROC: Receiver operating characteristic
S/T: Spontaneous/timed
TR: Thickening ratio
